# Impact of proton-beam irradiation on the electrical reliability and performance of LTPS and a-IGZO thin-film transistors

**DOI:** 10.1038/s41598-025-05664-z

**Published:** 2025-07-01

**Authors:** Junho Noh, Moonsoo Kim, Dongbhin Kim, Sungsoo Park, Hwan-Gyu Lee, Sungwoo Jung, Donghyun Kim, Nguyen Thanh Tien, Byoungdeog Choi

**Affiliations:** 1 Department of Electrical and Computer Engineering, Sungkyunkwan University, Suwon, South Korea; 2 Department of Semiconductor Convergence Engineering, Sungkyunkwan University, Suwon, South Korea; 3 Display Research Center, Samsung Display Company Ltd., Yongin, South Korea

**Keywords:** Radiation, Electrical reliability, Thin-film transistors (TFTs), Amorphous In–Ga–Zn–O (a-IGZO), Low-temperature polycrystalline silicon (LTPS), Density of state (DOS), Engineering, Nanoscience and technology

## Abstract

We investigate the impact of 5 MeV proton beam irradiation on the electrical reliability of low-temperature polycrystalline silicon (LTPS) and amorphous In–Ga–Zn–O (a-IGZO) thin-film transistors (TFTs). After irradiation, the threshold voltage (*V*_th_) of a-IGZO TFTs shifted from 0.31 to − 7.87 V, while field-effect mobility (*μ*_FE_) increased from 8.4 to 11.7 cm^2^/V∙s due to oxygen vacancy (V_o_) formation, enhancing channel conductivity. In contrast, LTPS TFTs exhibited severe degradation, with *V*_th_ shifting from − 3.18 to − 33.51 V and *μ*_FE_ dropping from 78.9 to 0.01 cm^2^/V s. Bias temperature instability tests showed significant deterioration in irradiated LTPS TFTs, whereas a-IGZO TFTs remained stable. This is attributed to the metastable a-IGZO lattice, which suppresses radiation-induced defect formation, whereas the LTPS lattice undergoes amorphization. X-ray Photoelectron Spectroscopy (XPS), and density of states (DOS) confirmed these mechanisms. Finally, we confirmed electrical performance recovery of irradiated TFTs through rapid thermal annealing (RTA) process. These findings provide insights into TFT degradation under radiation exposure and highlight the potential of a-IGZO and LTPS TFTs for radiation-hardened applications in radiography, military, aviation, and aerospace industries.

**Supplementary Information:**

The online version contains supplementary material available at 10.1038/s41598-025-05664-z.

## Introduction

Electronic devices operating in harsh radiation environments, such as deep space, aviation, aerospace, military, and nuclear facilities applications, are exposed to severe radiation-induced degradation. Cosmic radiation, primarily composed of high-energy protons, neutrons, and heavy ions, causes detrimental effects such as total ionizing dose (TID), displacement damage (DD), and single-event effects (SEE), which significantly impact the performance and reliability of electronics^1,2^. Protons, which constitute approximately 85% of cosmic radiation, are particularly responsible for malfunctions in spacecraft electronics, spacesuit-integrated flexible electronics, and display applications on the International Space Station (ISS)^3–6^. Understanding the interaction between semiconductor devices and radiation is therefore critical for developing radiation-hardened electronic applications in radiography, military, aviation, and aerospace industries^7,8^. Ahn et al. studied 5 MeV proton irradiation on β-Ga_2_O_3_ photodetectors and observed significant degradation in photocurrent and photoluminescence, attributed to deep-level defects acting as non-radiative recombination centers in the active region^9^. Pezzimenti et al. reported a performance degradation study of SiC vertical power MOSFETs under 3 MeV proton beam irradiation, observing reduced current, transconductance, and capacitance due to charge-carrier removal and defect generation in the epitaxial region^10^. Bonaldo et al. analyzed trench SiC power MOSFETs under X-ray and 3 MeV proton beam irradiation, observing strong TID sensitivity and DD-induced current degradation at > 10 Mrad due to defect formation in the SiC epitaxial layer^11^.

Amorphous oxide semiconductors (AOSs), such as amorphous In–Ga–Zn–O (a-IGZO), have emerged as promising materials due to their high carrier mobility (< 40 cm^2^/V s), low off-state current (*I*_off_), good uniformity, and compatibility with low-temperature processing (< 400 °C)^12,13^. Polycrystalline silicon (poly-Si) has also attracted significant attention owing to its high mobility and excellent electrical stability. Low-temperature polycrystalline silicon (LTPS) thin films are formed by recrystallizing amorphous silicon (a-Si) via excimer laser annealing (ELA), resulting in improved electrical reliability and performance^14^. Despite the inherent high *I*_off_, LTPS TFTs can achieve enhanced performance through off-bias stress techniques^15^. Owing to these properties, a-IGZO and LTPS thin films have been widely adopted as channel layers in advanced display technologies, such as low-temperature poly-Si and oxide (LTPO) displays and micro-light-emitting diode (μ-LED) displays. In particular, AOS materials are being explored for use in memory industry, such as cell transistors for 3-demenstional (3D) DRAM technology, due to their excellent electrical properties and process compatibility^16^. Given the increasing use of LTPS and a-IGZO TFTs in radiation-intensive environments, investigating their radiation-induced degradation and reliability issues is essential for developing robust semiconductor device technologies for extreme radiation exposure conditions.

This study examines the effects of 5 MeV proton beam irradiation on the electrical performance and reliability of a-IGZO and LTPS TFT devices. Electrical characteristics, including I–V characteristics and bias temperature instability stress test, were evaluated. X-ray photoelectron spectroscopy (XPS) was utilized to analyze radiation-induced chemical bonding changes, while defect states were quantitatively assessed using density of states (DOS) and oxide-trapped charge density (*N*_ot_) extraction from C–V characteristics before and after proton beam irradiation. Finally, recovery behavior of the degraded TFT performance under proton beam irradiation was confirmed via RT anneal in vacuum and an RTA process.

## Results and discussion

### Electrical characteristics of TFTs before and after proton beam irradiation

**Fig. 1:**
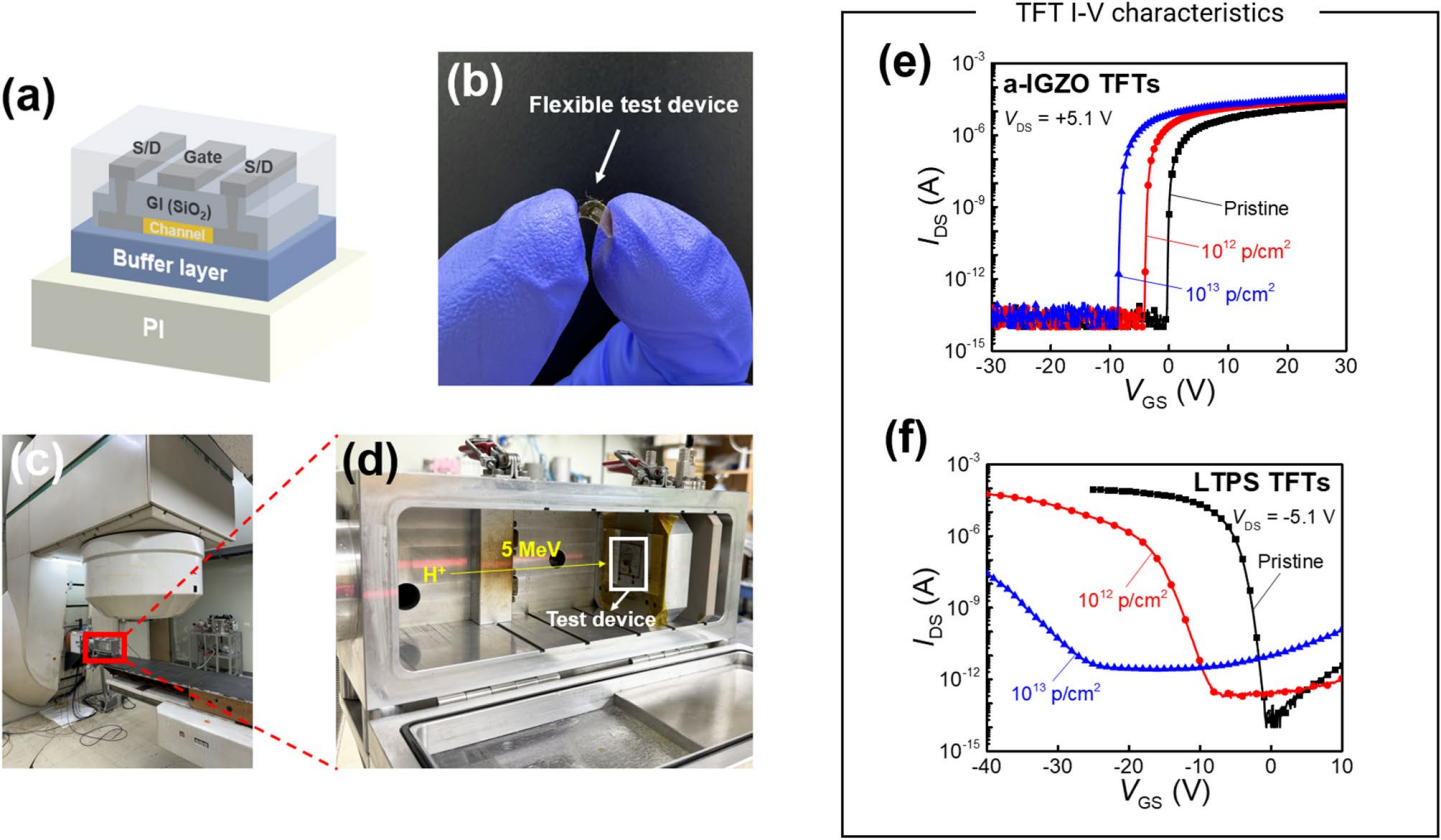
(**a**) Schematic diagram of TFT device structure (channel layers: LTPS, a-IGZO) and image of (**b**) a flexible test device used in this study. Images of (**c**) MC-50 cyclotron and (**d**) vacuum chamber with test device at the Korea Institute of Radiological and Medical Science (Republic of Korea) for proton beam irradiation test. Transfer curves of (**e**) a-IGZO and (**f**) LTPS TFTs under different proton beam irradiation dose levels (pristine, 10^12^, and 10^13^ p/cm^2^).

**Fig. 2:**
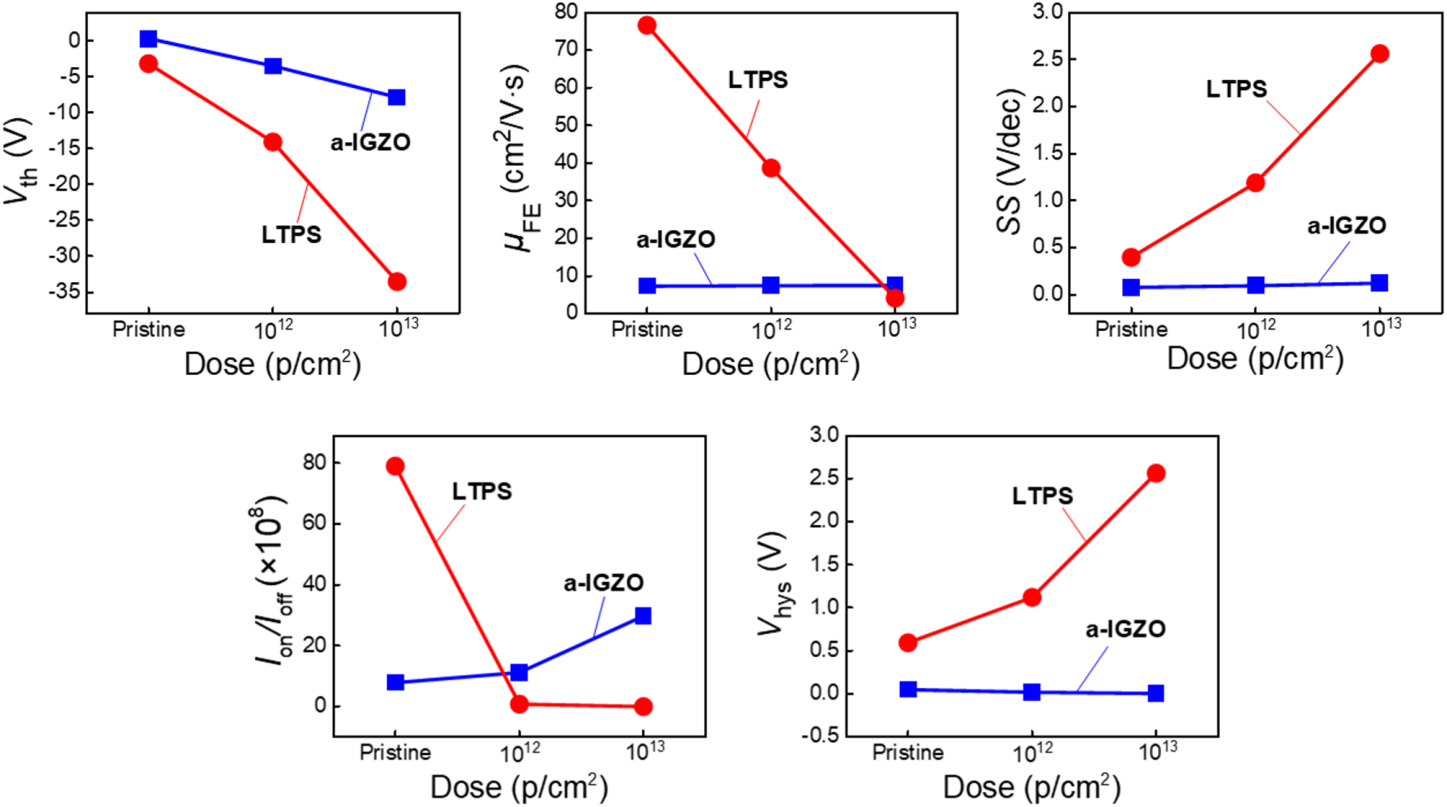
Electrical characteristic parameters comparison of LTPS and a-IGZO TFTs at different proton beam irradiation doses.

**Fig. 3:**
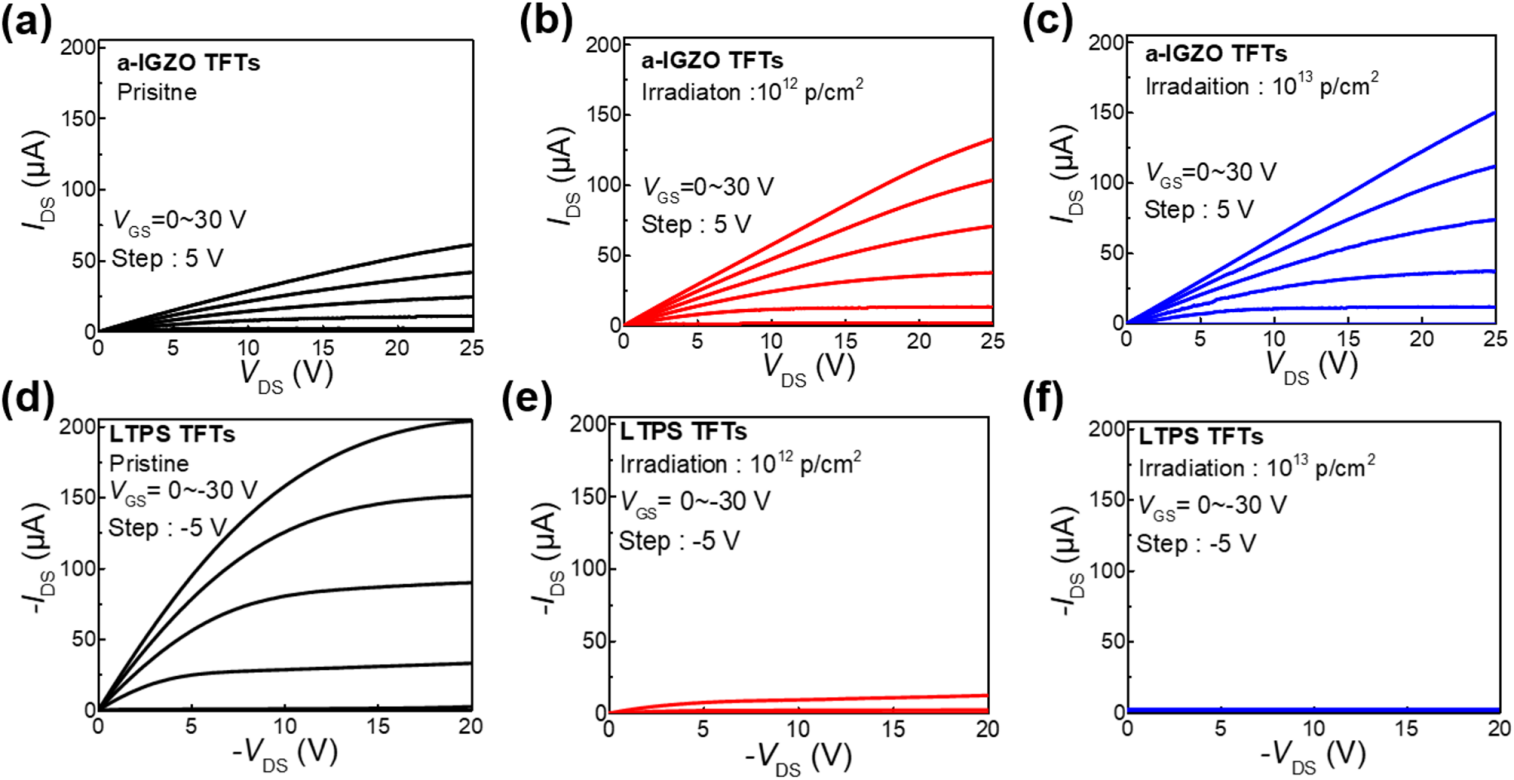
Output curves of (**a**), (**b**), (**c**) a-IGZO TFT and (**d**), (**e**), (**f**) LTPS TFT under different proton beam irradiation doses.

**Fig. 4:**
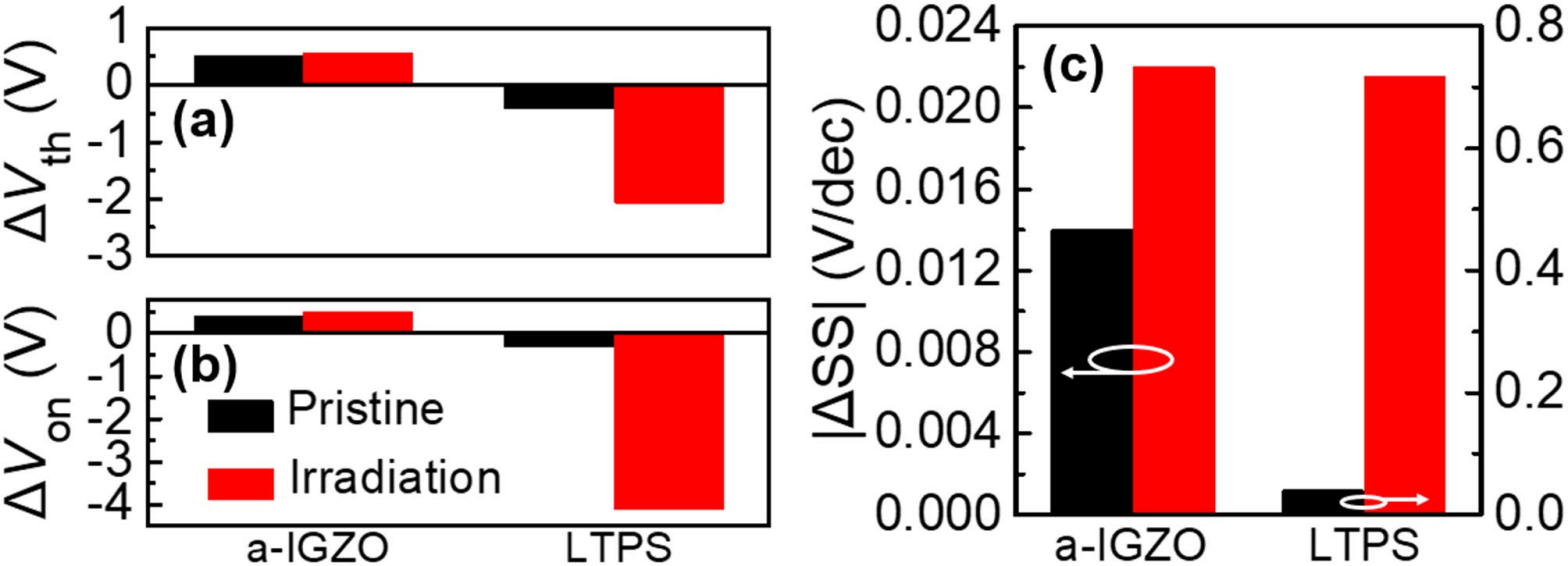
The electrical characteristics (Δ*V*_th_, Δ*V*_on_, and |ΔSS|) of pristine and irradiated a-IGZO and LTPS TFTs (10^13^ p/cm^2^) stressed under PBTS and NBTS, respectively. The bias instability tests were performed at 70 °C with *V*_GS_ =  ± 3 MV/cm for 3600 s.

Figure 1a and b show the top-gate structures of a-IGZO and LTPS TFTs fabricated on a polyimide (PI) substrate. Both devices share an identical TFT structure, differing only in their channel layers. Figure 1c and d depict the proton beam irradiation setup, where test devices were placed in a high-vacuum chamber to ensure complete energy transfer from the 5 MeV proton beam. The irradiation dose varied from 0 (denoted as pristine) to 10^12^ and 10^13^ at 5 MeV of beam energy and 10 nA of beam current. The selected 5 MeV proton energy and irradiation doses represent realistic and worst-case space-radiation conditions, corresponding to typical spectra observed in the Van Allen Belt and Low-Earth Orbit (100 keV–400 MeV)^17,18^. All electrical characteristics were extracted from a sample of 5 devices for each irradiation dose. Figure 1e and f show the transfer characteristics of pristine and irradiated a-IGZO and LTPS TFTs. Table 1 and Fig. 2 summarize the extracted electrical parameters. The *V*_th_ was obtained via the constant current method. The subthreshold swing (SS) was calculated at the subthreshold region of the transfer curve with the following equation: $\text{SS}=\frac{\partial {V}_{\text{GS}}}{\partial (\text{log}\left({I}_{\text{DS}}\right))}$. The on–off current ratio (*I*_on_/*I*_off_) was calculated as the ratio of the maximum drain-source current (*I*_DS_) in the on-state to its minimum value in the off-state. The *μ*_FE_ was extracted from the maximum transconductance at *V*_DS_ =  + 0.1 V for a-IGZO TFTs and − 0.1 V for LTPS TFTs. As shown in Fig. 1e, a-IGZO TFTs showed a substantial negative shift of *V*_th_ with increasing irradiation dose. At the same time, there was an increase in the *I*_DS_. The a-IGZO TFT had a *V*_th_ of − 0.31 V (pristine), which shifted to − 3.86 V (10^12^ p/cm^2^) and further to − 7.87 V (10^13^ p/cm^2^). The SS slightly increased 0.08 (pristine) to 0.10 V/dec (10^12^ p/cm^2^) and 0.13 V/dec (10^13^ p/cm^2^). These results indicate that the carrier conductivity of the a-IGZO channel was enhanced by proton beam irradiation. This implies that proton beam irradiation can induce an increase in V_o_, known to donate two free electrons within the a-IGZO channel layer^19^. Neutral oxygen vacancy (V_o_^0^) produced a deep donor state in the middle of the energy band gap near the valence band (*E*_v_) and generated electron–hole pairs. Ionized oxygen vacancies (V_o_^1+^, V_o_^2+^) produced a shallow donor-like state nearby the conduction band (*E*_c_) which assisted the excitation of electron in *E*_v_. Overall, the conductivity of AOS channels increased following the following mechanism: V_o_ → V_o_^2+^ + 2e^−^^20^. Despite the changes in SS and *I*_off_ values remained almost unchanged compared to their initial states, indicating the good stability of a-IGZO TFTs under proton beam irradiation. Figure 1f shows transfer curves of pristine and irradiated LTPS TFTs at doses of 10^12^ and 10^13^ p/cm^2^. The *V*_th_ shifted negatively from 0.4 V (pristine) to − 14 V (10^12^ p/cm^2^) and − 33.5 V (10^13^ p/cm^2^), indicating that irradiation induced significantly larger *V*_th_ shifts in LTPS TFTs than in a-IGZO TFTs. Furthermore, as shown in Fig. 1e, f and 2, the SS, *I*_on_/*I*_off_, and *μ*_FE_ of LTPS TFTs showed significant degradation after irradiation: the *I*_on_/*I*_off_ decreased from 78.9 × 10^8^ (pristine) to 0.81 × 10^8^ (10^12^ p/cm^2^) and 10^4^ (10^13^ p/cm^2^); the SS increased from 0.4 (pristine) to 1.19 (10^12^ p/cm^2^) and 2.56 (10^13^ p/cm^2^); and the *μ*_FE_ decreased from 76.4 cm^2^/Vs (pristine) to 38.7 cm^2^/V s (10^12^ p/cm^2^) and 2.18 cm^2^/V s (10^13^ p/cm^2^). The severe degradation of LTPS TFTs can be attributed to the radiation-induced formation of trap sites at the Poly-Si/SiO_2_ interface and within the LTPS channel. Additionally, an increase in the *N*_ot_ within the GI contributed to *V*_th_ shift and reliability instability of TFT devices^21^. The degradation in these electrical characteristics can be attributed to radiation-induced increases in the defect density at the Poly-Si/SiO_2_ interface, which hinder efficient channel formation. Additionally, radiation-induced defects can act as trap sites for conduction carriers, leading to significant decreases in SS, *I*_on_/*I*_off_, and *μ*_FE_^22^. The difference in degradation between a-IGZO and LTPS TFTs is attributed to the flexibility of amorphous lattice, which arises from their metastability and suppresses radiation-induced defect generation. In contrast, the LTPS lattice undergoes radiation-induced amorphization, leading to severe degradation^23,24^. Figure 3 illustrates output curves of a-IGZO and LTPS TFTs before and after proton beam irradiation. Figure 4a, b, and c illustrate output curves of the pristine and irradiated a-IGZO TFTs at different doses, showing an increase in *I*_DS_ with increasing irradiation dose. This substantiates that an increase in the total dose of proton beam irradiation can lead to an increase in V_o_, thereby enhancing the conductivity of a-IGZO channels and causing a negative shift in *V*_th_^25^. Figure 4d, e, and f show output curves of the pristine and irradiated LTPS TFTs at doses of 10^12^ and 10^13^ p/cm^2^, respectively. In contrast to a-IGZO TFTs, LTPS TFTs exhibited a significant decrease in *I*_DS_ with increasing irradiation dose due to radiation-induced lattice damage^24^. *V*_hys_ values of a-IGZO and LTPS TFTs were extracted after sweeping the gate voltage in the positive direction, followed by a subsequent reverse sweep in the negative direction. *V*_hys_ was typically due to defects at the channel/GI interface^26,27^. The *V*_hys_ of the a-IGZO TFT decreased from 0.05 V (pristine) to 0.02 V (10^12^ p/cm^2^) and 0.005 V (10^13^ p/cm^2^). In contrast, the *V*_hys_ of the LTPS TFT exhibited a notable increase from 0.59 V (pristine) to 1.12 V (10^12^ p/cm^2^) and 2.56 V (10^13^ p/cm^2^) (Supplementary Fig. S1). Proton beam irradiation induces an annealing effect via atomic collisions, generating localized heating inside the vacuum chamber. This effect promotes self-annealing in the a-IGZO TFTs, leading to reduced *V*_hys_. However, in LTPS TFTs, radiation damage dominates over self-annealing, resulting in increased *V*_hys_ and severe performance degradation^19,28^. These results showed that a-IGZO TFTs exhibited relatively higher resilience and stability under proton beam irradiation than LTPS TFTs, which exhibited significant degradation of TFT performance under the same irradiation test conditions. The difference in the degree of TFT degradation could be attributed to differences in material properties between amorphous and crystalline. X-ray diffraction (XRD) analysis revealed that the LTPS film exhibited reduced peak intensities at the (111), (220), and (311) planes after irradiation, indicating lattice disorder, whereas the a-IGZO film showed negligible change. These findings suggest superior structural stability of the a-IGZO channel under radiation (Supplementary Fig. S4)^19,29,30^.

**Table 1:** Electrical parameters extracted from the I–V characteristics at different proton beam irradiation doses.

	Dose (p/cm^2^)	*V*_th_ (V)	SS (V/dec)	*μ*_FE_ (cm^2^/Vs)	*I*_on_/*I*_off_	*V*_hys_ (V)
LTPS	Pristine	− 3.21 ± 0.06	0.40 ± 0.03	76.4 ± 0.23	78.9 ± 0.01	0.59 ± 0.03
	10^12^	− 14.1 ± 0.23	1.19 ± 0.06	38.7 ± 0.21	0.81 ± 0.03	1.12 ± 0.04
	10^13^	− 33.5 ± 0.32	2.56 ± 0.05	2.2 ± 0.09	0.01 ± 0.01	2.56 ± 0.05
a-IGZO	Pristine	− 0.31 ± 0.05	0.08 ± 0.02	8.4 ± 0.16	7.83 ± 0.02	0.05 ± 0.02
	10^12^	− 3.86 ± 0.18	0.10 ± 0.03	10.2 ± 0.24	11.2 ± 0.01	0.02 ± 0.01
	10^13^	− 7.87 ± 0.19	0.13 ± 0.02	11.7 ± 0.21	29.7 ± 0.03	0.005 ± 0.01

### Bias instability test before and after proton beam irradiation

Figure 4 asummarizes changes in electrical parameters after bias instability tests on pristine and irradiated a-IGZO and LTPS TFTs at an irradiation dose of 10^13^ p/cm^2^ (Supplementary Fig. S2). Positive bias temperature stress (PBTS) was applied to the a-IGZO TFTs at 70 °C with *V*_GS_ =  + 3 MV/cm for 3600 s. As for the LTPS TFTs, Negative bias temperature stress (NBTS) was applied at 70 °C with *V*_GS_ = − 3 MV/cm for 3600 s. The electrical parameters were analyzed to evaluate the impact of proton beam irradiation on TFT bias instability. The turn-on voltage (*V*_on_) was extracted as the *V*_GS_ at which the *I*_DS_ of TFTs started to increase sharply. As shown in Fig. 4a, the *V*_th_ of the pristine a-IGZO TFT shifted from 0.61 to 1.12 V (Δ*V*_th_ =  + 0.51 V), while that of the proton-irradiated a-IGZO TFT changed from − 7.39 V to − 6.81 V (Δ*V*_th_ =  + 0.57 V). In contrast, the LTPS TFT exhibited a *V*_th_ shift from − 2.9 V to − 3.3 V (Δ*V*_th_ = − 0.4 V) before irradiation, and a much larger negative shift from − 32.1 V to − 34.3 V (Δ*V*_th_ = − 2.2 V) after irradiation. As shown in Fig. 4b, the *V*_on_ of the pristine a-IGZO TFT increased from 0.0022 V to 0.41 V (Δ*V*_on_ =  + 0.41 V), and from − 8.05 V to − 7.53 V (Δ*V*_on_ =  + 0.52 V) after irradiation. In contrast, the pristine LTPS TFT showed a *V*_on_ shift from − 0.81 V to − 1.12 V (Δ*V*_on_ = − 0.31 V), and from − 21.6 V to − 25.7 V (Δ*V*_on_ = − 4.11 V) after irradiation. As shown in Fig. 4c, the SS of the pristine a-IGZO TFT increased from 0.112 V/dec to 0.126 V/dec (ΔSS =  + 0.014 V/dec), and from 0.141 V/dec to 0.163 V/dec (ΔSS =  + 0.022 V/dec) after irradiation. Meanwhile, the LTPS TFT exhibited more pronounced degradation, with SS increasing from 0.47 V/dec to 0.51 V/dec (ΔSS =  + 0.04 V/dec) in the pristine state, and from 1.99 V/dec to 2.71 V/dec (ΔSS =  + 0.72 V/dec) after irradiation.

These results showed that significant degradation in the bias stability of LTPS TFTs occurred after irradiation. In contrast, the bias instability of a-IGZO TFTs was robust at an irradiation dose of 10^13^ p/cm^2^. The superior reliability of a-IGZO TFTs can be attributed to their flexible chemical bond network, which allows structural disruptions to recombine and suppresses defect formation. In contrast, LTPS TFTs, with their rigid chemical bond network, underwent significant and permanent structural damage upon radiation exposure, leading to substantial deterioration in TFT electrical reliability^29,30^.

### XPS analysis for thin film characterization

**Fig. 5:**
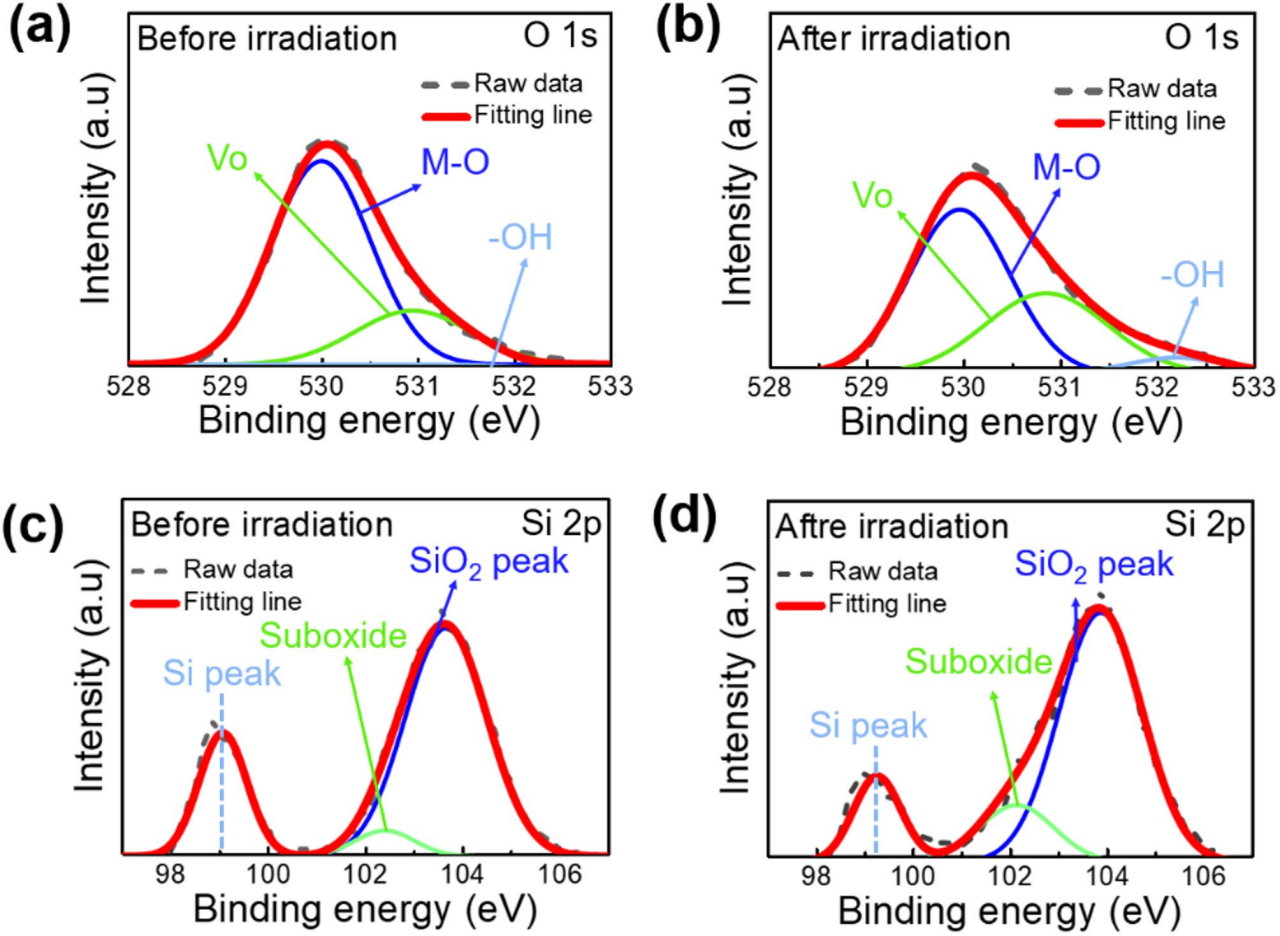
XPS results of (**a**) before (**b**) after O 1s spectra of a-IGZO thin films and (**c**) before (**d**) after Si 2p spectra of Poly-Si thin films at proton beam irradiation (dose : 10^13^ p/cm^2^).

Figure 5 presents XPS analysis results of pristine and irradiated a-IGZO and LTPS thin films (dose: 10^13^ p/cm^2^). Figure 5a and b compare the O 1s spectra of a-IGZO thin film. Figure 5c and d show Si 2p spectra at the Poly-Si/SiO_2_ interface. For the analysis of the a-IGZO thin film, deconvolution of the O 1s spectra revealed peak of M–O bonds at 530 ± 0.1 eV, oxygen vacancies (V_o_) at 531.2 ± 0.1 eV, and hydroxyl group (−OH) at 532 ± 0.5 eV. After irradiation, M–O bonds in the a-IGZO thin film decreased, while V_o_ and –OH peak intensity increased from 22 to 36% and from 2 to 6%, respectively. The decrease in M–O bonds was attributed usually to the weak bond of In–O being disrupted by energetic proton-induced atomic lattice displacement, causing oxygen loss and thus an increase in V_o_. At the same time, hydrogen present in the GI and buffer layers is incorporated into the a-IGZO channel layer, where it reacts with oxygen ions (O^2−^) via the following mechanism: H^+^ + O^2−^  → OH^−^  + e^−^. This reaction increases the –OH intensity and associated trap sites while simultaneously generating free electrons. This process is also enhanced by the localized annealing effect induced by proton beam irradiation^28,31^. Such increase can lead to enhanced conductivity within the a-IGZO channel^19^. As for the XPS analysis of the LTPS thin film nearby SiO_2_, deconvolution of the Si 2p spectra for LTPS thin film revealed Si peak, suboxide peak, SiO_2_ peak at 102.7 ± 0.1 eV, 103.2 ± 0.1 eV, 104 ± 0.1 eV, respectively. After irradiation, the Si peak in the LTPS thin film decreased, the suboxide peak intensity increased from 20 to 25%, and the SiO_2_ peak increased from 43 to 48%. Suboxide with dangling bonds can act as a defect at the nearby Poly-Si/SiO_2_ interface, trapping conduction carriers and significantly degrading the TFT reliability and performance^32^. This increase in suboxide peak was due to hole-induced cleavage of strained Si–O–Si bonds at the Poly-Si/SiO_2_ interface. As a result, proton beam irradiation can break Si–Si bonds in the Poly-Si/SiO_2_ interface, leading to a decrease in the Si peak and an increase in the SiO_2_ peak. The increase in the suboxide peak can also significantly affect the LTPS TFT performance^33,34^.

### Damage mechanisms under proton beam irradiation

**Fig. 6:**
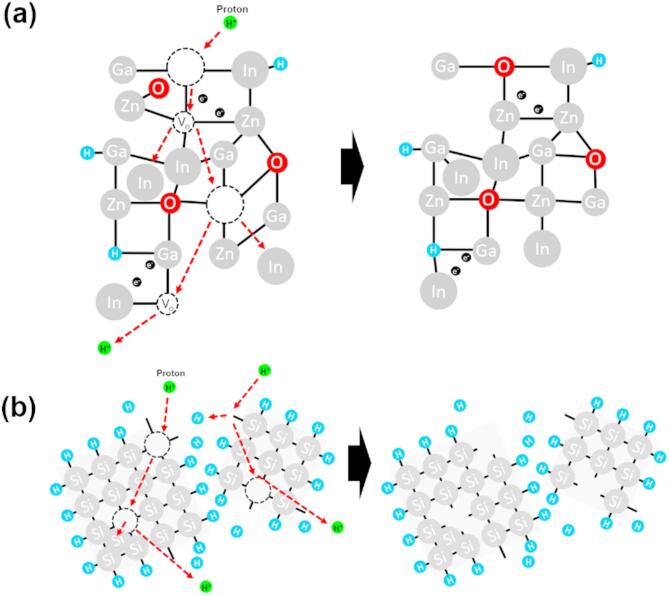
Schematic diagrams of proton beam irradiation induced damage mechanisms for (**a**) flexible bond network of a-IGZO channel (**b**) rigid bond network of LTPS channel.

Figure 6 illustrates the damage mechanisms of amorphous metal-oxide and polycrystalline silicon thin films under proton beam irradiation. In particle-based radiation, such as protons and neutrons, key factors influencing damage include energy, effective mass, and atomic collision probability. When energetic particles collide with an atomic lattice of materials, they induce atomic displacement cascades, where ejected atoms initiate further collisions. This cascade effect increases intrinsic defects in solid-state materials^29,30^. However, radiation-induced damage mechanisms differ significantly between amorphous and polycrystalline materials. Typically, an amorphous phase is more tolerant to radiation than a crystalline structure. Figure 6a illustrates the damage mechanisms in a-IGZO channels caused by proton beam irradiation. The amorphous phase has a structural chemical bond flexibility of amorphous lattice attributed to its metastability property. This flexible chemical bond structure allows the amorphous material to structurally rearrange damaged lattice atoms through collective atomic motion at the moment of radiation exposure, thereby suppressing the generation of radiation-induced defects. This structural atomic rearrangement typically requires enough activation energy. This energy is sufficiently supplied externally by the proton beam irradiation to allow rearrangement to occur in response to radiation damage^19,29,30^. It has been reported that the In_2_O_3_ matrix in the a-IGZO channel has a weaker binding energy than Ga_2_O_3_ and ZnO matrices, indicating that the loss of oxygen due to electric stress or external forces such as radiation occurs more easily in the In_2_O_3_ matrix than in Ga_2_O_3_ and ZnO matrices. When particles collide with the a-IGZO lattice, predominantly weak In-O bonds will break, leading to an increase in V_o_^19,35^. This results in a higher electron concentration within a-IGZO channels, substantially increasing the channel conductivity. Figure 6b illustrates the damage mechanisms in LTPS channels due to irradiation. LTPS channel layer contains Si–Si covalent bonds at grain and Si–H bonds at grain boundaries. This means that the LTPS channel layer has crystallinity on grains and a rigid chemical bond network structure that does not structurally rearrange the damaged lattice structure. When accelerated particles collide with this atomic rigid network in LTPS lattices, it causes permanent structural changes in grains and grain boundaries due to the cascade atomic displacement reactions of ejected atoms such as Si and H contents from grains and grain boundaries. This leads to increased permanent structural change and defects by radiation-induced amorphization of LTPS channel, thus degrading the TFT reliability and performance^36^. 5 MeV proton beam irradiation was found to fully penetrate the entire device stack and induce more damage in the a-IGZO channel region compared to the LTPS channel. Nevertheless, the a-IGZO TFT maintained superior structural integrity and electrical performance relative to the LTPS TFT, demonstrating its excellent radiation tolerance (Supplementary Fig. S4, S5, and S6).

### Radiation-induced defect characterization

**Fig. 7:**
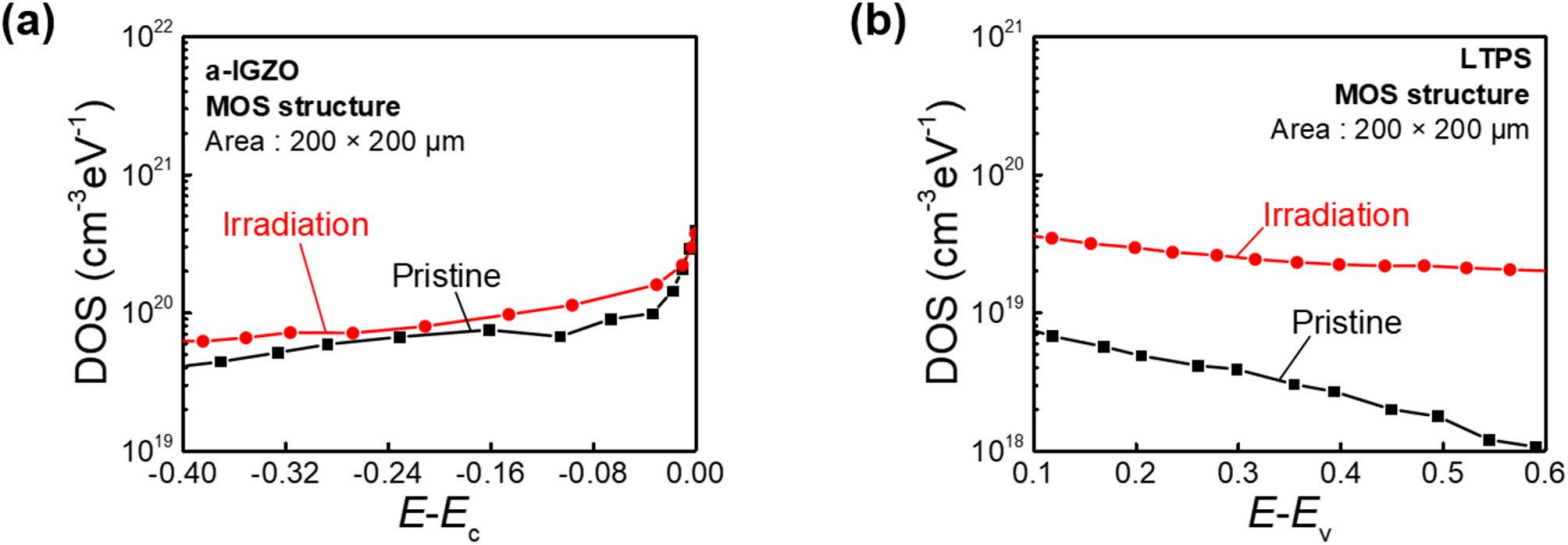
DOS distributions for defect quantification extracted of (**a**) a-IGZO and (**b**) LTPS MOS devices.

Defects in the channel and GI are major factors in the degradation of electronic device performance and reliability under ionizing radiation exposure. Ionizing radiation generates electron–hole pairs in the GI, some of which become trapped, while trap sites are generated at the interface. These effects cause *V*_th_ shifts, an increase in SS, and significantly impact the long-term reliability of devices^37–39^. Figure 7a and b shows DOS distributions of a-IGZO and LTPS MOS devices extracted using a conductance method, respectively. For both devices, the x-axis represents the energy level extracted from surface potential (*ψ*_S_). The y-axis represents trap densities quantitatively as a function of energy level. Trap sites for both devices were determined with the following equation: *D*_it_ = $(\frac{{G}_{\text{p}}}{\omega })$_peak_, Where *G*_p_ denoted conductance and *ω* denoted the angular frequency. The *ψ*_S_ for extraction of energy level was determined from the following equation: *ψ*_S_ = ${\int }_{{V}_{FB}}^{{V}_{GS}}1-\frac{{C}_{GS}}{{C}_{OX}}d{V}_{\text{GS}}$, where *V*_FB_ denoted flat band voltage, and *C*_GS_ and *C*_ox_ denoted gate-source capacitance and oxide capacitance per unit area, respectively^40^. The a-IGZO MOS was extracted before and after irradiation (10^13^ p/cm^2^). The LTPS MOS device was analyzed before and after irradiation at 10^12^ p/cm^2^. However, at 10^13^ p/cm^2^, severe degradation caused an extreme *V*_FB_ shift, making the C–V curve exceed the measurement range of the LCR meter. In Fig. 7a, the a-IGZO MOS showed a slight increase in trap density after proton beam irradiation, which was attributed to the increase in hydrogen incorporation into a-IGZO channel and V_o_ after proton beam irradiation. This supports the increase in conductivity and negative *V*_th_ shift of a-IGZO TFTs under proton beam irradiation^25^. On the other hand, from Fig. 7b, LTPS MOS showed a significant increase in value of trap densities despite being measured at a lower irradiation dose than a-IGZO MOS. These effects cause an increase in SS and a degradation in TFT reliability. *N*_ot_ for a-IGZO and LTPS MOS devices under proton beam irradiation were determined with the following equation: *N*_ot_= $\frac{{C}_{ox}\Delta {V}_{FB}}{q}$^40–42^. The *N*_ot_ for a-IGZO MOS increased from 5.6 × 10^8^ cm^−2^ at an irradiation dose of 10^12^ p/cm^2^ to 9.6 × 10^8^ cm^−2^ at 10^13^ p/cm^2^. For LTPS MOS, *N*_ot_ increased to 1.5 × 10^9^ cm^−2^ at 10^12^ p/cm^2^. However, C–V data at 10^13^ p/cm^2^ was unavailable due to a significant shift in *V*_FB_ after proton beam irradiation, which was out of the measurement range (Supplementary Fig. S3). While *N*_ot_ increased in both types of device upon radiation exposure, LTPS device exhibited greater sensitivity to *N*_ot_ increase compared to a-IGZO device. This suggests that LTPS-based devices are more vulnerable to performance degradation and reduced reliability under proton beam irradiation than a-IGZO-based devices.

### Recovery of radiation-induced defects in TFTs

**Fig. 8:**
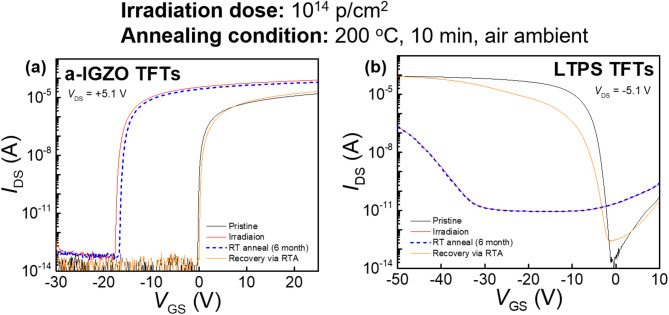
Transfer curves before and after proton beam irradiation at a dose of 10^14^ p/cm^2^, along with the recovery of TFT performance through RT annealing and RTA processes for (**a**) a-IGZO and (**b**) LTPS TFTs.

Figure 8a and b present the transfer curves of a-IGZO and LTPS TFTs before and after proton beam irradiation at a dose of 10^14^ p/cm^2^. Following irradiation, RT annealing was conducted over six months to facilitate the recovery of radiation-induced defects. This process involved storing the irradiated TFT devices in a vacuum desiccator with floating electrodes at RT. During RT annealing, oxide and interface trap charges evolved, leading to a gradual restoration of the electrical characteristics of the TFT devices over time. Additionally, an air-ambient RTA process was performed at 200 °C for 10 min to further enhance the recovery of electrical properties. After proton beam irradiation, a-IGZO TFTs exhibited increased SS values, unlike those observed at irradiation doses of 10^12^ p/cm^2^ or 10^13^ p/cm^2^. However, RT annealing resulted in a partial recovery of the electrical properties. This suggests that under high-energy, high-dose irradiation conditions, radiation-induced defect generation dominates over the defect suppression attributed to the a-IGZO lattice flexibility, causing electrical degradation in a-IGZO TFTs. Nevertheless, defects gradually recover over time, leading to a partial restoration of electrical characteristics^30^. Following the RTA process, the a-IGZO TFTs fully recovered to their initial transfer characteristics due to oxide and interface defects being thermally eliminated. In contrast, as shown in Fig. 8b, LTPS TFTs experienced severe electrical degradation due to radiation-induced defect formation at the Poly-Si/GI interface and within the GI, which is attributed to the non-flexibility of the LTPS lattice. Unlike a-IGZO TFTs, LTPS TFTs did not fully recover after RTA. This discrepancy arises from differences in radiation-induced defect formation, which are closely related to the distinct chemical bond network structures of amorphous and polycrystalline channel layers, as well as their different applications in TFT technology^29,30^.

## Conclusion

In this study, we investigated the effects of high-energy proton beam irradiation on the electrical reliability of a-IGZO and LTPS TFTs fabricated on a flexible substrate. Our findings reveal that LTPS TFTs suffered severe radiation-induced degradation, with *V*_th_ shifting from − 3.18 to − 33.51 V, SS increasing from 0.4 to 2.56 V/dec, and *μ*_FE_ decreasing from 76.4 cm^2^/V s to 0.01 cm^2^/V s. This degradation is attributed to the rigid chemical bonding network of the LTPS channel, which is unable to suppress radiation-induced defect formation. In contrast, a-IGZO TFTs exhibited greater radiation tolerance, demonstrating significantly less sensitivity to radiation-induced degradation. This resilience is due to the amorphous nature and flexibility of the a-IGZO lattice, which enables structural rearrangement and suppression of defect generation upon radiation exposure. As a result, a-IGZO TFTs maintained stable electrical performance and reliability under high-energy proton beam exposure. These results suggest that a-IGZO TFTs are promising candidates for radiation-hardened electronics in aviation, aerospace, radiography, and other radiation-intensive environments.

### Experimental

#### Device fabrication

Both p-type LTPS and n-type a-IGZO TFTs used a top-gate structure on a coplanar substrate with a 250 nm SiO_2_ buffer layer deposited on a PI substrate via plasma enhanced chemical vapor deposition (PECVD). For LTPS TFTs, amorphous silicon (a-Si) thin film was deposited via PECVD, recrystallized by XeCl (λ = 308 nm) ELA, and patterned for the channel layer. Activation annealing was performed at 350 °C. A 150 nm SiO_2_ GI was deposited, followed by a 200 nm Mo source/drain electrode via DC sputtering. For a-IGZO TFTs, a 30 nm a-IGZO channel layer was deposited via radio frequency sputtering, followed by activation annealing at 300 °C for 1 h in an N_2_ atmosphere. A 150 nm SiO_2_ GI was deposited, and a 200 nm Mo source/drain electrode was added. A 200 nm SiN_x_ passivation layer was deposited on both TFTs. Both devices had W/L = 8/8 μm. Metal − oxide − semiconductor (MOS) capacitors were fabricated using the same process, with a 200 × 200 μm Mo contact area.

#### Device failure test under proton beam irradiation

a-IGZO and LTPS TFTs and their thin films were exposed to a proton beam using a MC-50 cyclotron for proton beam irradiation (Korea Institute of Radiological and Medical Science (KIRAMS), Republic of Korea). The total dose irradiation varied from 0 (denoted as pristine), 10^12^ and 10^13^ p/cm^2^ at 5 MeV of beam energy with 10 nA of beam current. No electrical bias was applied to any terminals of devices during proton beam irradiation.

#### Electrical and thin film characterization

Electrical measurements were performed for pristine TFTs, followed by measurements of electrical properties after each dose of 10^12^ and 10^13^ p/cm^2^. Electrical characteristics of the TFT device were measured on the day of proton beam irradiation using an Agilent 4156C in a dark ambient at room temperature. For a-IGZO TFTs, transfer curves were measured at *V*_GS_ = − 30 ~ 30 V at *V*_DS_ =  + 5.1 V. Output curves were measured at *V*_DS_ = 0–25 V, *V*_GS_ = 0 ~ 30 V (*V*_GS,step_ =  + 5 V). For the LTPS TFT, transfer curves were measured at *V*_GS_ = − 40 ~ 10 V at *V*_DS_ = − 5.1 V and output curves were measured at *V*_DS_ = 0 ~ − 20 V, *V*_GS_ = 0 ~ − 30 V (*V*_GS,step_ = − 5 V). All electrical characteristics were extracted from a sample of 5 devices for each irradiation dose. Both a-IGZO and LTPS thin films were irradiated with a dose of 10^13^ p/cm^2^. Chemical bonding state changes of LTPS and a-IGZO thin films induced by proton beam irradiation were observed via XPS using a PHI 5000 VersaProbe instrument (ULVAC PHI, Japan). The C 1s peak was used to calibrate all core level spectra of each thin film.

## Electronic supplementary material

Below is the link to the electronic supplementary material.

Supplementary Material 1

## Data Availability

The datasets analysed in this study are available from the corresponding authors upon reasonable request.
