# Supplementary material for: Impact of proton-beam irradiation on the electrical reliability and performance of LTPS and a-IGZO thin-film transistors
Source: Sci Rep. 2025 Jul 1;15:20435. doi: 10.1038/s41598-025-05664-z (PMC12215767; doi:10.1038/s41598-025-05664-z)
Supplement: Supplementary file 1 — Supplementary Material 1 [file 41598_2025_5664_MOESM1_ESM.docx]

**Supplementary Information**

**Impact of Proton-Beam Irradiation on the Electrical Reliability and Performance of LTPS and a-IGZO Thin-Film Transistors**

*Junho Noh^1^, Moonsoo Kim^1^, Dongbhin Kim^1^, Sungsoo Park^1^, Hwan-Gyu Lee^1^,
Sungwoo Jung^2^, Donghyun Kim^2^, Nguyen Thanh Tien^3^, and Byoungdeog Choi^1,2*^*

^1^Department of Electrical and Computer Engineering, Sungkyunkwan University, Suwon, South Korea.

^2^ Department of Semiconductor Convergence Engineering, Sungkyunkwan University, Suwon, South Korea.

^3^ Display Research Center, Samsung Display Company Ltd., Yongin, South Korea.

*Corresponding author: bdchoi@skku.edu (B.D. Choi)


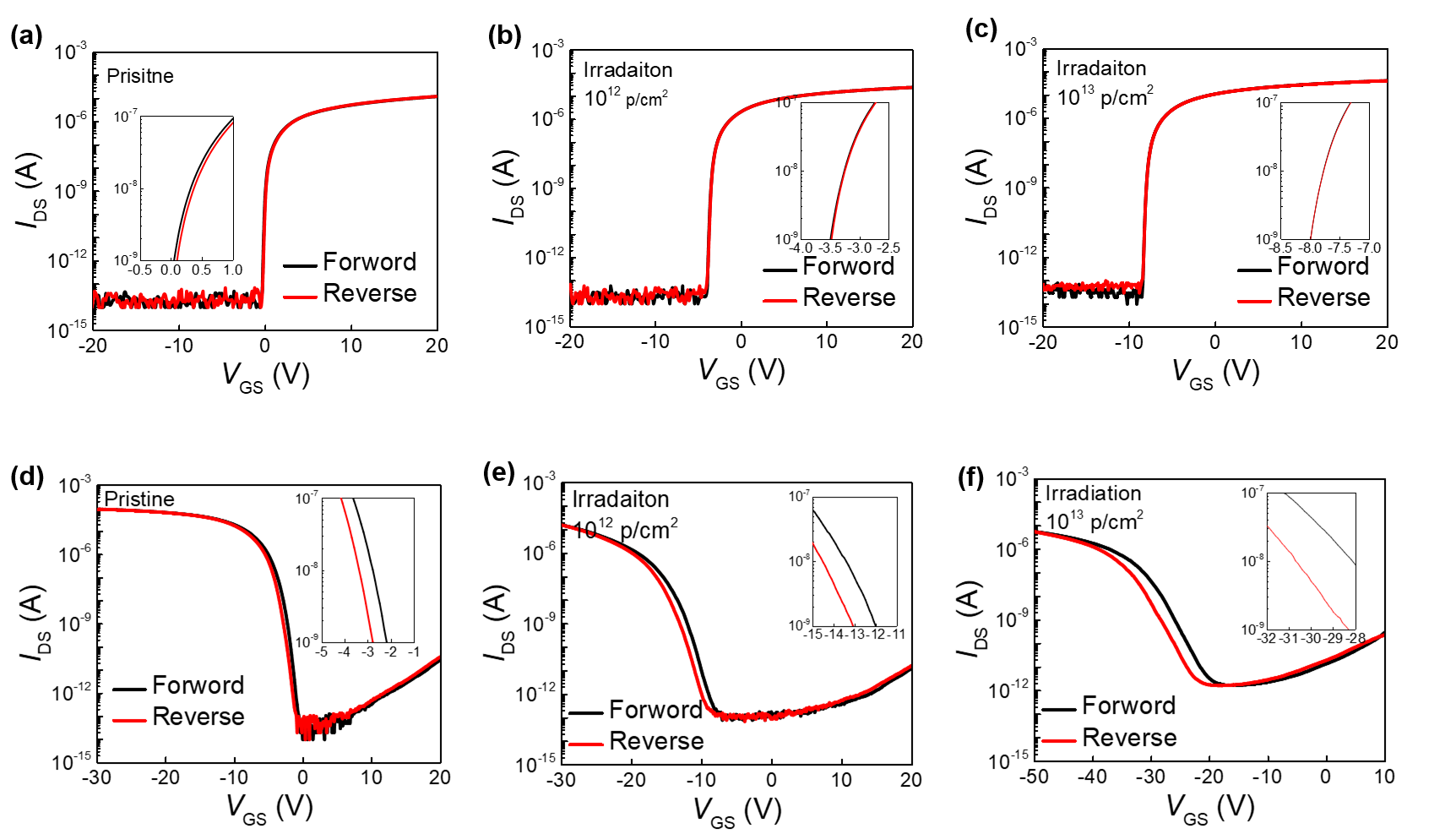


**Fig. S1.** Hysteresis of transfer curves for (**a**), (**b**), and (**c**) a-IGZO TFTs, and (**d**), (**e**), and (**f**) LTPS TFTs under different proton beam irradiation doses.
As the irradiation dose increases, the *V*_hys_ of a-IGZO TFTs decreases due to the annealing effect, while LTPS TFTs show an increasing trend.


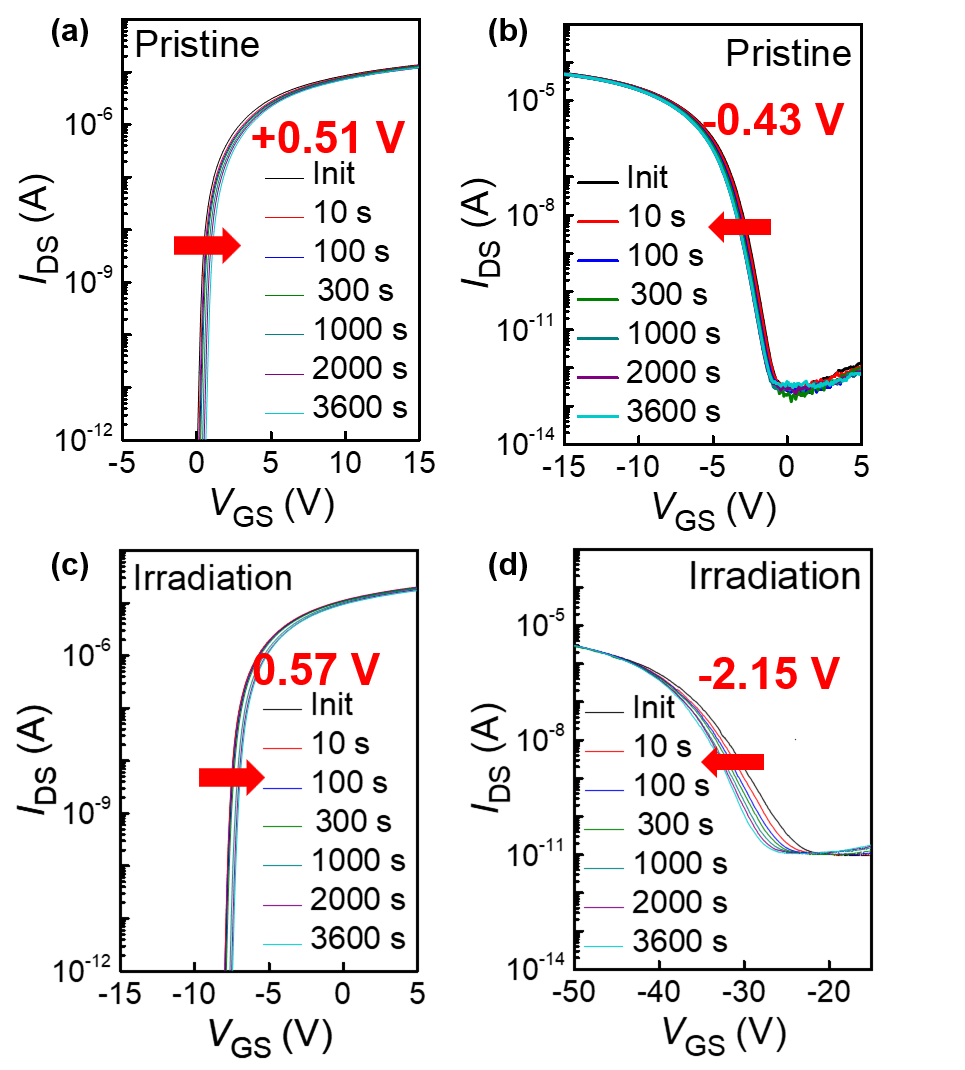


**Fig. S2.** Results of bias temperature instability tests before and after proton beam irradiation at a dose of 10^13^ p/cm^2^ for (**a**), (**c**) a-IGZO TFTs and (**b**), (**d**) LTPS TFTs. a-IGZO TFTs show no significant bias instability induced by irradiation, in contrast to LTPS TFTs.


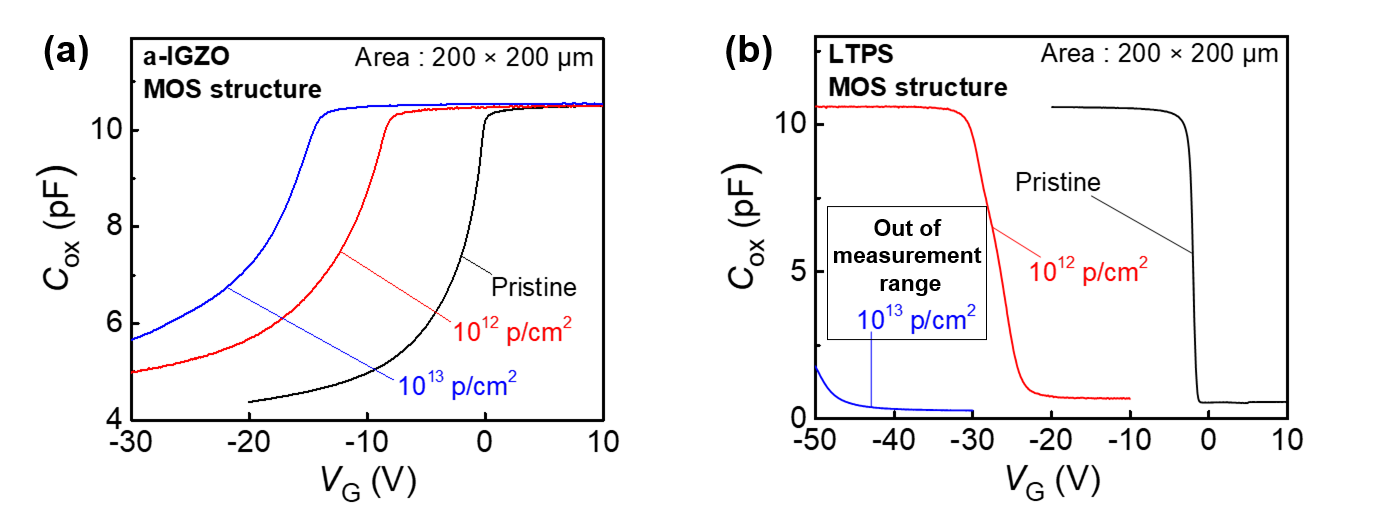


**Fig. S3.** C−V curves of (**a**) a-IGZO and (**b**) LTPS MOS devices under different proton beam irradiation doses. Unlike a-IGZO devices, LTPS devices exhibit significant degradation in C−V characteristics due to proton beam irradiation, showing increased susceptibility to radiation. As a result, the 10^13 p/cm² dose exceeds the measurement range of the LCR meter.


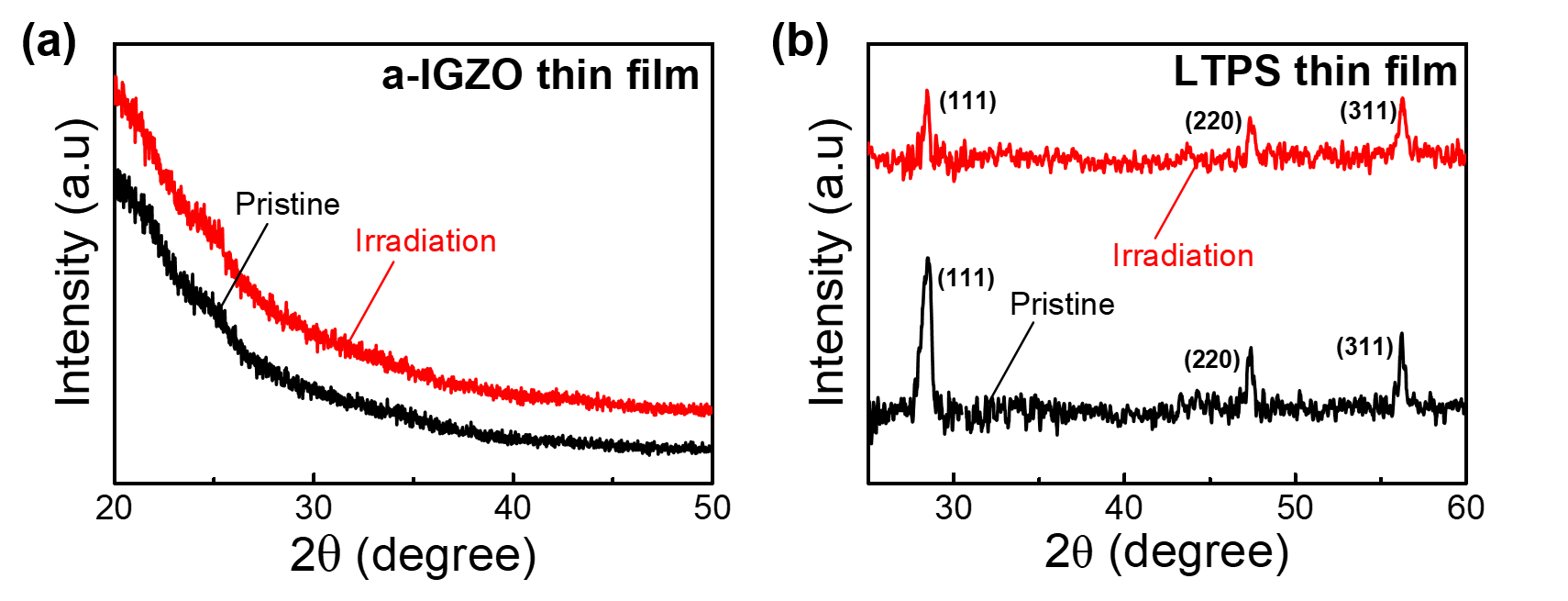


**Fig. S4.** XRD results of (**a**) a-IGZO and (**b**) LTPS thin films before and after proton beam irradiation at a dose of 10^13^ p/cm^2^.
No phase transition was observed in the a-IGZO thin film after irradiation. In contrast, the intensity of the (111), (220), and (311) peak in the LTPS thin film significantly decreased, indicating amorphization compared to the pristine LTPS thin film.

**
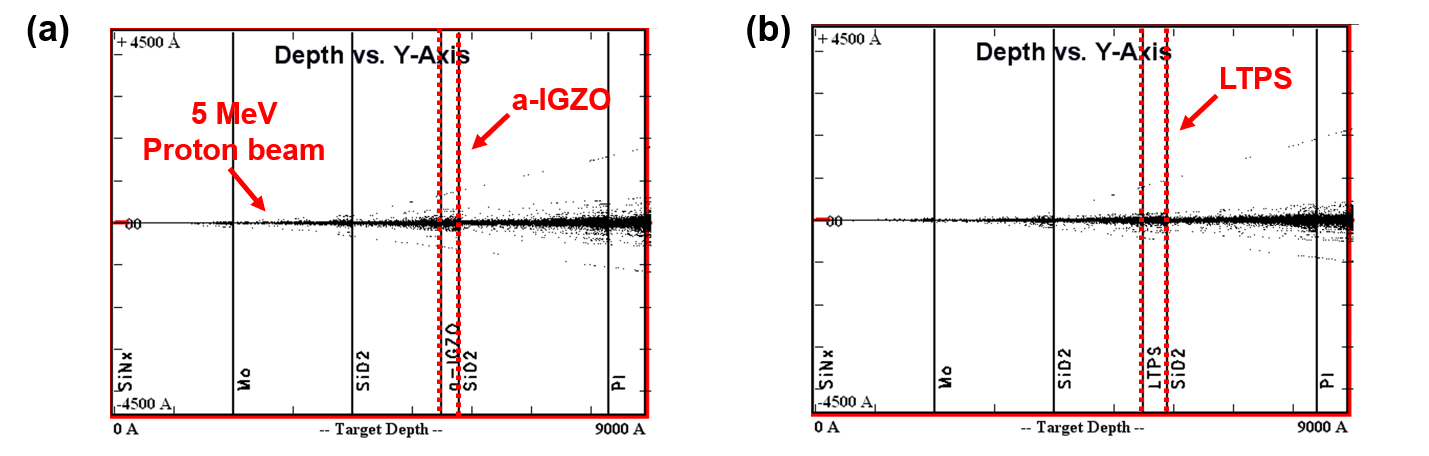
**

**Fig. S5.** SRIM simulation results showing the proton penetration depth profiles (depth vs. Y-axis) for the multilayer structure of SiNx/Mo/SiO₂/channel/SiO₂/PI, where the channel layer consists of (a) a-IGZO and (b) LTPS thin films, after 5 MeV proton beam irradiation.
No phase transition was observed in the a-IGZO thin film after irradiation. In contrast, the intensity of the (111), (220), and (311) peak in the LTPS thin film significantly decreased, indicating amorphization compared to the pristine LTPS thin film.


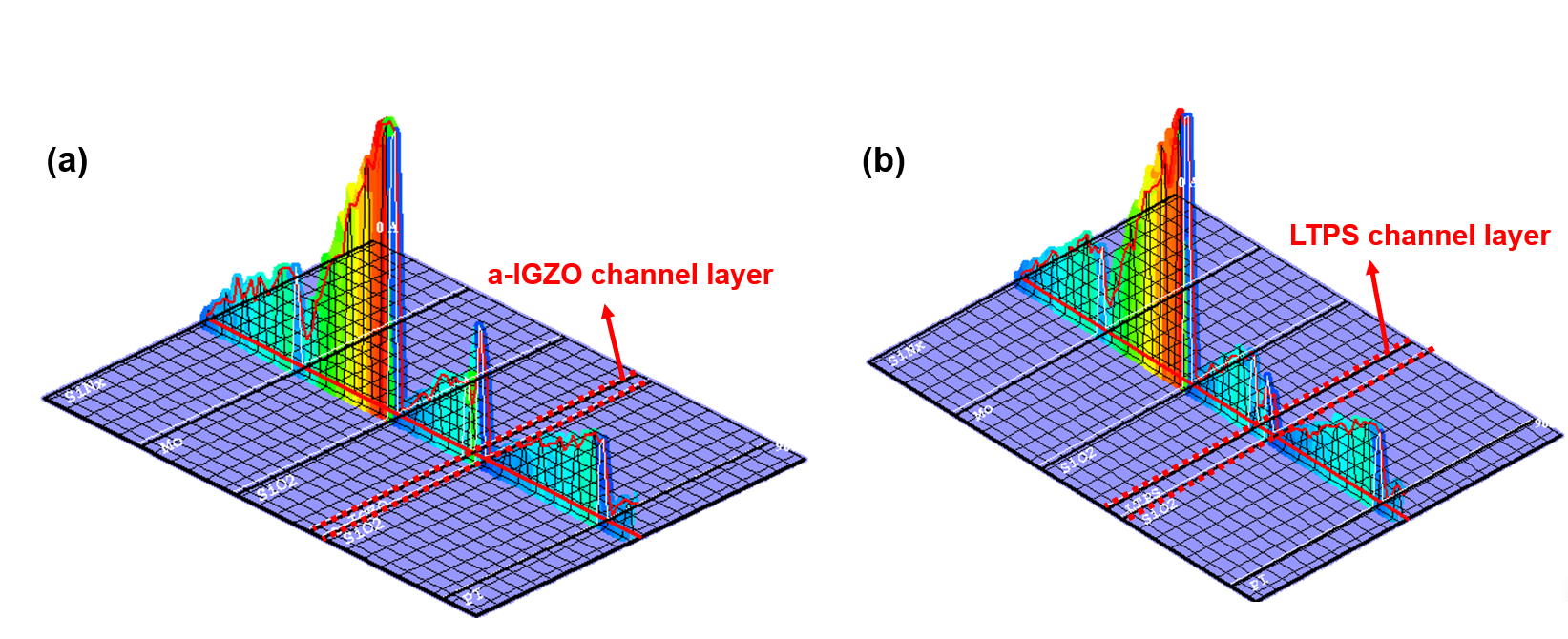


**Fig. S6.** SRIM simulation results showing displacement damage distributions in (a) a-IGZO and (b) LTPS thin films after 5 MeV proton beam irradiation.
Due to the higher material density of a-IGZO compared to LTPS channel, a-IGZO channel is subject to greater collision events under 5 MeV proton beam irradiation, as confirmed by the SRIM simulation results.
